# Vaping epidemic among the youth in Pakistan: urgent measures required to combat the rising trend

**DOI:** 10.2144/fsoa-2024-0011

**Published:** 2024-05-15

**Authors:** Shahzaib Samad, Bilqees Baloch, Muhammad Abdul Qadeer

**Affiliations:** 1 Jinnah Sindh Medical University, Rafiqi HJ Shaheed Road Karachi, 75510, Pakistan; 2 Ziauddin Medical College, 4/B, Shahrah-e-Ghalib, Block 6, Clifton Karachi, Sindh, 75600, Pakistan

**Keywords:** addiction, electronic smoking, epidemic, Pakistan, vaping, youth

The history of the vape device, also known as an electronic cigarette, dates back to its manufacture in Beijing in 2003, followed by its introduction to the US market in 2007 [1]. The device operates using a lithium battery, a heating coil, and a reservoir for liquid chemicals, which deliver a controlled dose of nicotine in vapor form [1]. Nicotine, a catecholamine with sympathomimetic effects, rapidly enters the bloodstream and crosses the blood–brain barrier within seconds when inhaled [2]. Initially promoted as a safer alternative to traditional cigarettes, vaping was intended to counteract the harmful effects of smoking, while also providing mood enhancement and a sense of euphoria [3]. However, the detrimental effects of nicotine vaping far outweigh its limited benefits. This commentary delves into the concerning surge of youth vaping in Pakistan, with a specific emphasis on the prevalence, the associated health risks, and the factors contributing to the rise in adolescent vaping. Additionally, in this article, we explore potential solutions to mitigate the incidence of vaping.

## Vaping prevalence & health risks among adolescents

The global e-cigarette and vape market size was valued at US $22.45 billion in 2022 and is expected to grow at a compound annual growth rate of 30.6% from 2023 to 2030 [4]. It is experiencing tremendous growth in the West and Southeast Asian countries such as Bangladesh, Nepal, India and Pakistan, Pakistan is emerging as a major vaping business hub. It is projected that the revenue from e-cigarettes will reach $77.2 million by 2024 in Pakistan, with an annual growth rate of 1.39% [5]. The popularity of vaping in Pakistan has been on the rise for several years. A cross-sectional study conducted in 2017 by medical students in Karachi revealed that many participants were using vaping devices without fully understanding their contents and harmful effects [6].

Recent studies have revealed that the additive chemicals in e-liquids have potential deleterious effects on the body, its aerosols induce DNA damage, decrease antioxidant defenses and negatively affect cell viability and proliferation. Acrolein, also a component, exhibits cytotoxicity, cross-links DNA and inhibits enzymes. Reactive aldehydes induce oxidative stress, contributing to cardiovascular, respiratory, pulmonary and oral diseases. E-liquid constituents, irrespective of nicotine, affect gingival fibroblasts and oropharyngeal mucosa, and induce oxidative stress. Components like cinnamaldehyde in e-cig liquids are immunosuppressive. Overall, vaping use poses significant health concerns for oral tissues as well as the immune system [7].

## A gateway to drugs

A recent study has revealed a notable connection between baseline e-cigarette use and the resurgence of marijuana consumption among individuals with a history of marijuana use, characterizing e-cigarette use as a gateway to marijuana reinitiation. Beyond the widely recognized concern that e-cigarettes may act as a gateway to marijuana, there exists a potential for e-cigarette use to reintroduce marijuana consumption among those with prior marijuana experience [8]. In terms of health risks, a substantial majority (around 80%) of reported cases of e-cigarette or vaping product-associated lung injuries were associated with THC-containing e-cigarettes, while approximately 58% involved the use of nicotine-containing products alongside THC or cannabinoids [9]. Although cannabis e-cigarettes tainted with vitamin E acetate were primarily linked to EVALI, cases of acute lung injuries related to cannabis inhalation were documented before 2019, raising concerns about other cannabis components or additives being responsible. Despite these ongoing issues, the introduction of novel cannabis vaporizer ingredients, such as Δ8-tetrahydrocannabinol, Δ10-tetrahydrocannabinol, tetrahydrocannabinol and cannabichromene, further complicates the landscape [10]. To effectively address safety concerns associated with cannabis e-cigarettes and vaping, a comprehensive understanding of the latest products, delivery modes and ingredients is essential.

## A call for action

The prevalence of vaping in Pakistan has the potential to lead to a catastrophic situation, similar to the past acceptance and heavy advertisement of cigarettes. Tobacco companies once reassured the public that cigarettes were harmless, and it took years for the dangers to be realized [11]. A similar trend is now emerging with vaping, and by the time its side effects are understood, it may have already become an epidemic in Pakistan. Few restrictions on vaping, including its use, advertisement, promotion, sponsorship and packaging, make it readily available to a wider population [12]. The availability of flavored liquids among vaping products in Pakistan makes it especially appealing to the youth, who are the primary target market. Other factors such as peer pressure, the absence of social stigma and easy accessibility contribute to its popularity among young people. This poses a perplexing conundrum for the nation, as it grapples with rampant inflation and must now address this pressing issue [13]. Efforts are being made worldwide to tackle the vaping crisis. China, for instance, has banned the sale of flavored e-cigarettes as part of a comprehensive crackdown on the industry [14]. Similarly, India implemented a complete ban on electronic smoking in 2019 [15]. According to WHO, 34 countries have banned the sale of e-cigarettes, while 88 countries do not have a minimum age for purchasing e-cigarettes and 74 countries have no regulations in place for these harmful products [16]. Pakistan lies in the latter group of countries and therefore, could learn from the actions of countries like India and China.

The solution to this dilemma lies with the government and individuals who are committed to bringing about change. The government should launch awareness campaigns, revise policies to impose a ban on vaping in public places, impose higher tariffs on such products and decrease accessibility of teenagers to it. Additionally, the government should mandate that companies list the ingredients on vaping product labels, enhancing consumer awareness of the associated hazards.

Individually, we can initiate and support social media campaigns to educate people about the drawbacks of vaping. As members of the medical community, we can play a crucial role by informing patients of the health risks associated with it and providing assistance to those who are already addicted. Since there are limited studies on this matter, there also needs to be greater emphasis on longitudinal studies done by researchers to see the long-term effects of vaping.
